# physician assisted dying : A French Perspective - a new revolution?

**DOI:** 10.1192/j.eurpsy.2024.118

**Published:** 2024-08-27

**Authors:** P. Courtet

**Affiliations:** University of Montpellier, Montpellier, France

## Abstract

**Abstract:**

The debates on euthanasia and assisted suicide (EAS) are topical in Europe. The extension of EAS for psychiatric reasons, already legalized in some countries, raises ethical and clinical issues, given the proximity between suicidal patients and patients who request or have accessed EAS. How can ESA be reconciled with the promotion of suicide prevention, which kills nearly 10,000 people per year in France? We will raise here several key questions that deserve a clear answer before considering going further in the social debates: how to ensure the irreversibility of psychological suffering? how to ensure that patients requesting EAS have full decision-making capacity? how to judge therapeutic futility? It seems crucial to protect the most vulnerable patients by ensuring that psychiatry benefits from scientific progress and can offer new solutions to suffering patients.

Thes issues will be discussed viewing the proposed law on EAS in France, which is supposed to come in February 2024…

**Disclosure of Interest:**

None Declared

